# Imaging of a retained laparotomy towel that migrated into the colon lumen

**DOI:** 10.4103/0971-3026.54889

**Published:** 2009-08

**Authors:** Umut Ozyer, Fatih Boyvat

**Affiliations:** Department of Radiology, Faculty of Medicine, Baskent University, Ankara, Turkey

**Keywords:** Retained sponge, surgery complications, transmural migration

## Abstract

Retention of surgical instruments, most commonly small laparotomy sponges, is a known complication of surgery. Such retained instruments may remain silent or may cause a variety of complications. We report a case in which a retained laparotomy towel migrated into the colon. This is an infrequently reported complication. We were able to document the passage of the towel through the colon on plain radiographs. The USG and MRI findings are also described.

## Introduction

Migration into the bowel lumen is an infrequent complication seen with retained laparotomy sponges.[1,2] The ileum is the most common part of the intestine into which migration takes place, followed by the jejunum and duodenum.[1] We report a case of colonic migration of a retained large laparotomy towel without significant obstruction. We were able to document its journey from the transverse to the sigmoid colon, until its extraction with a sigmoidoscope.

## Case Report

**Figure 1:(A-C):**
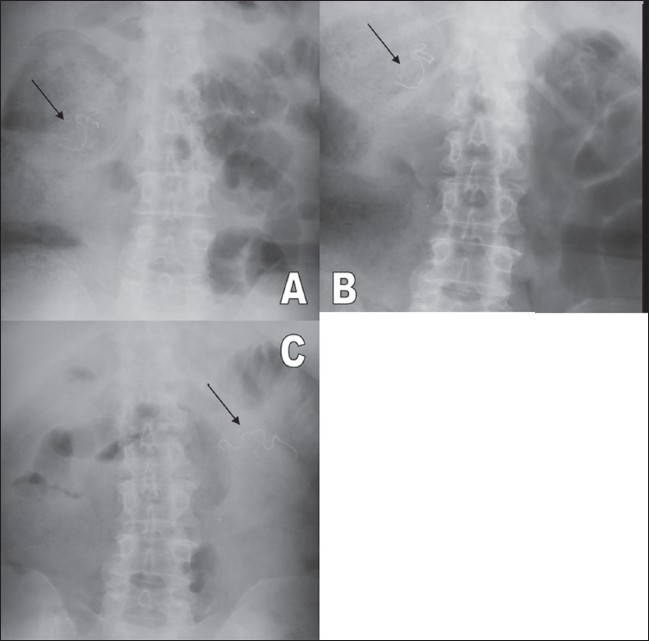
Plain abdominal radiographs show migration of the radiopaque marker (black arrows) from the right upper quadrant (A), moving upwards towards the center (B) and then to the left upper quadrant (C)

**Figure 2:**
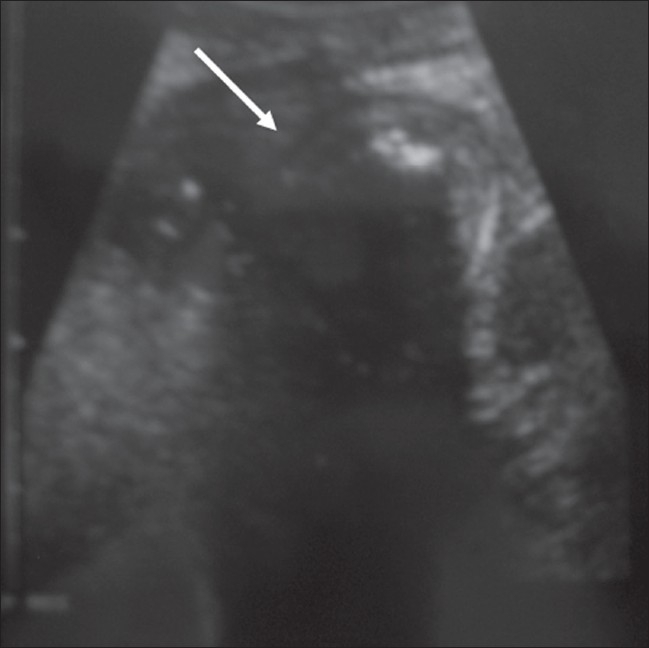
USG shows a mass with distinct borders (arrow) and an echogenic component with strong acoustic shadowing within the mass

**Figure 3:**
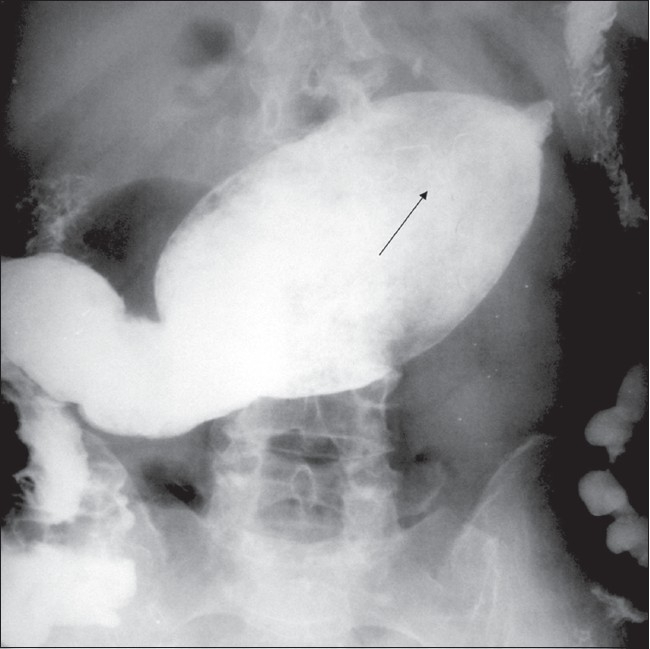
Barium enema shows dilation of the transverse colon by a mass, with the faintly detectable opacity of the marker (arrow). There is no dilatation proximal to the mass, while the distal passage is diminished

**Figure 4:**
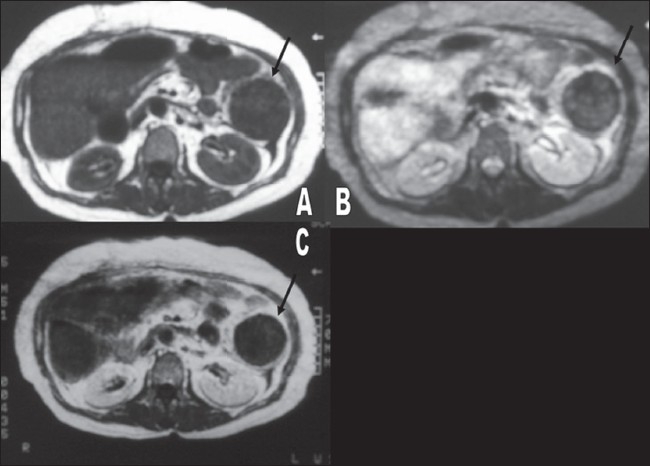
MRI of the abdomen shows a hypointense mass with scattered intensities within the colonic lumen (arrows) on T1W (A) and fat-saturated T2W (B) images. A hyperintense rim surrounds the mass on the T2W image. After gadolinium injection, a fat-saturated T1W image (C) shows rim enhancement with no difference in mass intensity

**Figure 5:**
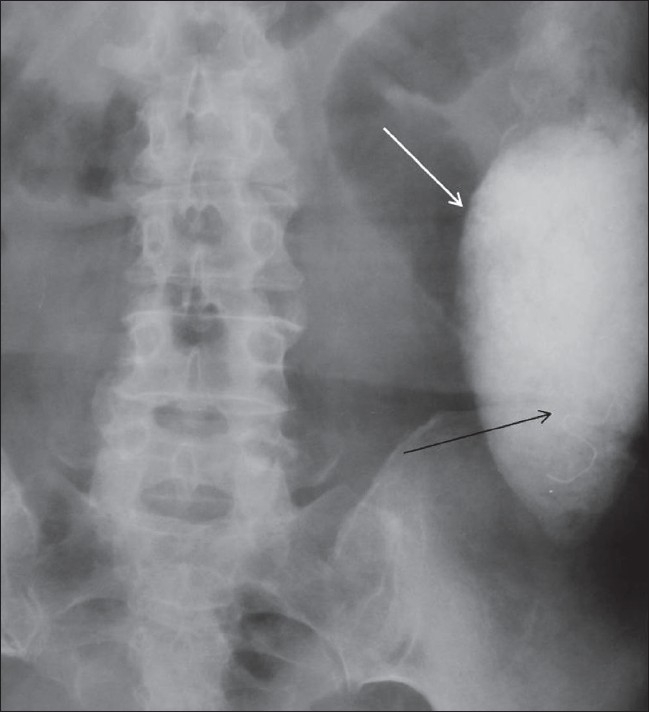
Plain radiograph obtained 6 h after barium enema shows the mass opacified with barium (white arrow); the marker has migrated inferiorly towards the left lower quadrant (black arrow)

A 58-year-old woman underwent a partial cholecystectomy at a local hospital for gall bladder perforation following acute cholecystitis. As per the postsurgical notes, the surgeons were unable to perform a cholecystectomy and so they drained the abscess and sutured the rest of the gall bladder. Her recovery was uneventful and she was discharged after a month. Three months later she was readmitted to the same hospital with abdominal pain, nausea, and vomiting. Physical examination was unremarkable, apart from mild abdominal tenderness. She had mild leukocytosis of 11,000/μl. On review, we found that her first plain abdominal radiograph showed a linear, curved radiopacity resembling the marker of a sponge in the right side of the abdomen at the level of the second lumbar vertebra [Figure 1A]. Another radiograph obtained later on the same day showed slight displacement of the marker to the level of the twelfth thoracic vertebra [Figure 1B]. This was not considered significant at the time. Six days later the patient was referred to our clinic after a third plain abdominal radiograph showed displacement of the marker to the left upper quadrant [Figure 1C]. USG revealed a curvilinear hyperechoic structure within the intestinal lumen, with intense posterior acoustic shadowing [Figure 2]. A single-contrast barium enema study showed that the transverse colon on the left side was dilated and the faint relative opacity of the marker could be distinguished within [Figure 3], though an obvious filling defect was not seen. It was not easy to exclude bezoar or a calcified mass and so MRI was ordered after the enema. We did not opt for CT because barium had just been used. On MRI [Figure 4], we detected a 25 × 6 × 5.5-cm well-circumscribed ovoid mass located in front of the left kidney and within the descending colon. It was hypointense on both pulse sequences, with some scattered and wavy intensities within. Surrounding the mass, there was an irregular hyperintense rim on the T2W images, which turned hypointense on T1W images and enhanced after intravenous gadolinium administration. This may have been due to the inflammation around the hypointense sponge. A radiograph obtained the same day after the MRI showed a mass coated with barium and containing the marker within, the marker having reached the rectosigmoid junction [Figure 5]. We decided to extract the mass endoscopically and, the next day, a mass containing a 30 × 30-cm laparotomy towel was extracted with a sigmoidoscope.

## Discussion

Being the most common retained surgical instrument, there are many reported complications of retained sponges, e.g., peritonitis, adhesions, abscess formation, vessel occlusion, etc. An unusual complication is migration into the gastrointestinal tract lumen and consequent intestinal obstruction; alternatively, it may be extruded via the rectum, as happened in our case.[1–3]

Since 1940, there have been only 24 cases reported of transmural migration of surgical sponges into the gastrointestinal tract.[1–5] To the best of our knowledge, there have been only five cases where the retained sponge was found in the colon; in two of these cases the sponge was extracted via endoscopy as in our case. In two cases, the retained sponge migrated into the transverse colon and reached the rectum by peristalsis. The other reported cases followed pelvic surgeries, and the sponge migrated only a short distance into the sigmoid colon or rectum. In our case, we were able to document the movement of a 30-cm long towel (larger than in any previously reported case), propelled by peristalsis from the transverse to the sigmoid colon.[1,2,4,6,7]

Plain radiographs are the easiest way to identify a retained sponge with a radiopaque marker. However, even in the presence of the radiopaque marker, it is possible to over-read or under-read sponge presence, as happened initially in our patient. The sponge may also show peripheral calcification and a whorl-like appearance, but this was not observed in our case. A migrated sponge may show up as an intraluminal filling defect in a dilated loop of intestine and, in later images, may be seen as a barium-coated lesion with strands and air bubbles within.

On USG, a retained sponge is seen as an echogenic mass with dense acoustic shadowing or, less commonly, as a cystic mass containing curvilinear and strongly hyperechoic content.[5] MRI appearances can vary. Granuloma formation around the retained sponge presents as an inhomogeneous mass that is slightly hyperintense to muscle on T1W and T2W images. An amorphous hyperintense center and wavy low-signal structures are possible on T2W images.[3,8] A hyperintense periphery and hypointense center have also reported.[9] This variability is possibly due to variation in the time interval between the surgery and the examination. In the early stages, a hyperintense area of inflammation surrounding the hypointense sponge is visualized on T2W images. The fibrotic capsule is hypointense on all pulse sequences, whereas the central necrotic area becomes hyperintense over time.[8,9]

In conclusion, when a retained laparotomy sponge has been diagnosed on plain radiographs, radiologists should keep in mind that transmural migration into the gastrointestinal system is possible. Barium enema, USG, CT, and MRI help in identifying the exact location and thus aid in selecting the best treatment option.

## References

[1] Cruz RJ, Poli de Figueiredo LF, Guerra L (2003). Intracolonic obstruction induced by a retained surgical sponge after trauma laparatomy. J Trauma.

[2] Choi JW, Lee CH, Kim KA, Park CM, Kim JY (2006). Transmural migration of surgical sponge evacuated by defecation: Mimicking anintraperitoneal gossypiboma. Korean J Radiol.

[3] Lo CP, Hsu CC, Chang TH (2003). Gossypiboma of the leg: MR imaging characteristics: A case report. Korean J Radiol.

[4] Godara R, Marwah S, Karwasra RK, Goel R, Sen J, Singh R (2006). Spontaneous transmural migration of surgical sponges. Asian J Surg.

[5] Yeung KW, Chang MS, Huang JF (2004). Imaging of transmural migration of a retained surgical sponge: A case report. Kaohsiung J Med Sci.

[6] Klein J, Farman J, Burrell M, Demeter E, Frosina C (1988). The forgotten surgical foreign body. Gastrointest Radiol.

[7] Manabe T, Goto H, Mizuno S, Kanematsu M, Hoshi H (1997). A case of retained surgical sponge penetrated into the sigmoid colon. Nippon Igaku Hoshasen Gakkai Zasshi.

[8] Mochzuki T, Takehara Y, Ichijo k, Nishimura T, Takahashi M, Kaneko M (1992). Case report: MR appearance of a retained surgical sponge. Clin Radiol.

[9] Boyvat F, Saatçi I, Özmen MN, Çekirge HS (1995). Retained sponge in the neck: MR appearance. AJNR Am J Neuroradiol.

